# Recognition of Oxidized Lipids by Macrophages and Its Role in Atherosclerosis Development

**DOI:** 10.3390/biomedicines9080915

**Published:** 2021-07-29

**Authors:** Nataliya V. Mushenkova, Evgeny E. Bezsonov, Varvara A. Orekhova, Tatyana V. Popkova, Antonina V. Starodubova, Alexander N. Orekhov

**Affiliations:** 1 Pharmstandard Ventures, 10 Testovskaya Street, 123112 Moscow, Russia; mushenkova@mail.ru; 2 Laboratory of Angiopathology, Institute of General Pathology and Pathophysiology, 125315 Moscow, Russia; evgeny.bezsonov@gmail.com (E.E.B.); a.h.opexob@gmail.com (A.N.O.); 3 Institute of Human Morphology, 117418 Moscow, Russia; 4 V.A. Nasonova Institute of Rheumatology, 115522 Moscow, Russia; popkovatv@mail.ru; 5 Federal Research Centre for Nutrition, Biotechnology and Food Safety, 109240 Moscow, Russia; avs.ion@yandex.ru; 6 Therapy Faculty, Pirogov Russian National Research Medical University, 117997 Moscow, Russia

**Keywords:** atherosclerosis, LDL, oxidized LDL, macrophage, inflammation, immunomodulation

## Abstract

Atherosclerosis is a multifactorial chronic disease that has a prominent inflammatory component. Currently, atherosclerosis is regarded as an active autoimmune process that involves both innate and adaptive immune pathways. One of the drivers of this process is the presence of modified low-density lipoprotein (LDL). For instance, lipoprotein oxidation leads to the formation of oxidation-specific epitopes (OSE) that can be recognized by the immune cells. Macrophage response to OSEs is recognized as a key trigger for initiation and a stimulator of progression of the inflammatory process in the arteries. At the same time, the role of oxidized LDL components is not limited to pro-inflammatory stimulation, but includes immunoregulatory effects that can have protective functions. It is, therefore, important to better understand the complexity of oxidized LDL effects in atherosclerosis in order to develop new therapeutic approaches to correct the inflammatory and metabolic imbalance associated with this disorder. In this review, we discuss the process of oxidized LDL formation, mechanisms of OSE recognition by macrophages and the role of these processes in atherosclerosis.

## 1. Introduction

The multifactorial nature of atherosclerosis pathogenesis makes studying this disease challenging. According to current view, atherosclerosis can be considered as a chronic inflammatory disease associated with progressive accumulation of lipids and inflammatory cells in the arterial wall. The atherogenic process begins with deposition of low-density lipoprotein (LDL), which is normally present in the blood plasma, in the subendothelial space of the arterial wall. In human arteries, such deposition often occurs at the sites of laminar flow perturbation, where activated or dysfunctional endothelial cells are present [1].

For many years, LDL was known as the main source of lipid accumulation in atherosclerosis, and much of anti-atherosclerotic therapies is aimed at correcting the blood lipid profile to slow down the disease progression. However, native (non-modified) LDL is not able to induce lipid accumulation in cultured arterial wall cells [2]. Therefore, a mere increase of circulating LDL level cannot explain the disease pathogenesis. Careful analysis of LDL fractions obtained from the blood of atherosclerotic patients revealed that LDL particles can undergo multiple atherogenic modifications that alter their physical-chemical properties. These multiple modifications occur in course of a cascade of physical-chemical transformations, the earliest of which is desialylation (loss of sialic acid residues) [3,4,5,6]. Desialylation is followed by the reduction of LDL particle size, increase of its negative charge, increase of LDL particle density, delipidation and LDL oxidation, which results in an increase of apolipoprotein B (apoB)-bound cholesterol [4,7]. The available results suggest that LDL oxidation may not be the initial step of LDL modification, but rather occurs in specific LDL fractions that are already modified; hence, it can be distinguished from native LDL, such as small dense LDL (sdLDL) and electronegative LDL. Furthermore, it was not possible to detect artificially introduced oxidized LDL (oxLDL) in the blood, although multiply modified LDL particles obtained from circulating blood were found to be oxidized [8]. It is also possible that LDL oxidation does not occur in the circulation, but instead takes place in the vascular wall. Despite the described controversies, oxLDL proved to be an important model for studying intracellular cholesterol accumulation and many other aspects of atherosclerosis pathogenesis.

Inside the arterial wall, oxLDL provides for oxidation-specific epitopes (OSE) that can be recognized by the innate immune system cells as damage-associated molecular patterns (DAMPs) [9]. These early events trigger the immune response, which eventually involves many cellular subtypes of both innate and adaptive immunity [1]. It was shown that macrophages along with T lymphocytes are the major subset of inflammatory cells in atherosclerotic lesions [10]. Moreover, phagocytic macrophages appear to be a major cell type responsible for intracellular lipid accumulation in atherosclerosis. A recent study of the transcriptome of LDL-induced macrophages demonstrated that native LDL and oxLDL changed the expression of different sets of genes [11]. Macrophages internalize oxidized lipoproteins via macropinocytosis, phagocytosis and receptor-mediated uptake. These processes are mediated by two main classes of membrane receptors involved in the sensing of oxLDL: scavenger receptors (SRs) and Toll-like receptors (TLRs), although soluble receptors may also be important for atheroinflammation progression and atherosclerosis complication development [12,13,14]. 

Macrophages, the key players of the innate immune system, have been identified as one of the cell types that directly respond to the presence of modified atherogenic LDL, including oxLDL. Following oxLDL recognition and internalization, macrophages undergo metabolic and functional reprogramming [11]. This process involves decrease of phagocytic activity, increase of pro-inflammatory cytokines production, and differentiation of macrophages into foam cells. Excessive foam cell formation results in the appearance of fatty streaks, the first grossly visible stage of atherosclerotic lesion. OxLDL was also described as an important driver of pro-inflammatory (also known as M1) macrophage polarization [11]. Pro-inflammatory macrophages interact with T-cells to drive the inflammatory response and atheroma progression, presenting antigens to T cells activating T-helper type-1 (Th1) responses. In turn, Th1 cells stimulate pro-inflammatory activation of macrophages by creating a specific cytokine environment inside the growing plaque. 

In order to improve anti-atherosclerosis therapies, it is important to better understand the signaling effects of oxLDL, mediated by its interaction with macrophages, which represent the key responding population that orchestrates different mechanisms of atherosclerosis plaque initiation, progression and rupture. At the same time, possible anti-inflammatory signaling properties of oxLDL should not be neglected, since they may be important for tissue reparation and inflammation resolution. It this work, we discuss both aspects of oxLDL interaction with immune cells and the role of such interactions in atherosclerosis.

## 2. Pathways of Lipid Oxidation and Their Relevance for Atherosclerosis

The presence of activated endothelial vascular cells, neutrophils, macrophages and T and B cells in atherosclerotic plaques, together with the proinflammatory cytokine environment, suggests that atherosclerosis is an active immunopathological process [1]. The hypothesis of oxLDL acting as a trigger of atherosclerosis development originated from the studies in 1980s–1990s that showed that macrophages treated with oxLDL, but not native LDL, accumulated cholesterol esters [15]. Another study demonstrated the presence of autoantibody response to oxLDL in apolipoprotein E deficient mice [16]. Since then, it has become clear that atherosclerosis is an autoimmune process and oxidized forms of LDL are among most validated autoantigens relevant to atheroinflammation [1]. However, the details of atherogenic modification of LDL are still being elucidated. Studies of LDL fractions extracted from the plasma of atherosclerosis patients suggested that oxidation of LDL particles is preceded by a cascade of multiple modifications, starting from desialylation and followed by particle delipidation, reduction of particle size, acquisition of electric charge and an increase of density. This process is accompanied by the loss of antioxidant agents from the LDL particle. It was shown that multiple-modified LDL contained 1.5–2-fold less antioxidants, including coenzyme-Q10, beta-carotene, alpha- and gamma-tocopherols and lycopene. Moreover, higher susceptibility of multiple-modified LDL was demonstrated in vitro [8].

**Figure 1:**
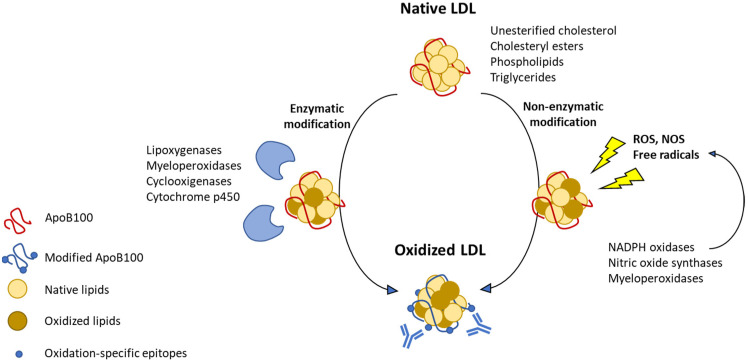
Mechanisms of lipids oxidation by peroxide generated through two main pathways.

The size of LDL particles ranges from 18 to 25 nm. The particle contains a single Apolipoprotein B100 (ApoB100) molecule and a lipid part consisting of unesterified cholesterol, cholesteryl esters (CE), phospholipids (PL) and triglycerides (TG). CE, PL and TG are all esters incorporating fatty acyl chains. Common non-saturated fatty acyls present in LDL are linoleic, arachidonic and docosahexaenoic acids, which are common polyunsaturated fatty acyls (PUFA) in these esters. The term oxLDL is broad and includes various oxidative changes to LDL lipid moieties as well as to ApoB100. Lipid oxidation is initiated by the removal of a hydrogen from the CH2 group that separates -C=C– units in PUFA, so called bis-allylic hydrogen, with the addition of oxygen radical [17]. Both enzymatic and non-enzymatic pathways of lipids peroxide generation are known (Figure 1).

Enzymatic pathways may involve activities of lipoxygenases (LO), myeloperoxidases (MPO), cyclooxygenases and cytochrome P450. The resulting products vary depending on the PUFA substrate and the enzyme involved and include hydroxyeicosatetraenoic acids, leukotrienes, lipoxins, hydroxyoctadecadienoic acids, resolvins in the case of lipoxygenase oxidation, prostaglandins, prostacyclin, thromboxanes in the case of cyclooxygenase oxidation or epoxyeicosatrienoic acids, 20-hydroxyeicosatetraenoic acid, thromboxanes and prostacyclins as a result of P450 activity. There may be species-specific differences in the level of expression of individual enzymes, which can influence LDL oxidation and atherosclerosis progression. For example, it was shown that murine atherosclerotic lesions contained virtually no traces of MPO, in contrast to human atheroma, and murine leukocytes contained >10- to 20-fold less MPO per cell than found in humans [18]. Therefore, although MPO deficit in mice has no effect on atheroinflammation, human MPO transgenic mice have accelerated atherosclerotic plaque development [18]. The pathogenic role of MPO in human atherosclerosis was revealed by the EPIC/Norfolk study on >25,000 healthy individuals, showing the prospective risk of developing symptomatic coronary heart disease positively correlated with baseline MPO levels [19]. 

Non-enzymatic mechanisms of LDL oxidation are mediated by free radicals that are generated mainly by NADPH oxidases and nitric oxide synthases in the presence of transition metal ions (Fe^2+^ and Cu^2+^). Myeloperoxidase can also initiate non-enzymatic lipid peroxidation through the generation of reactive oxygen species (ROS). Non-enzymatic peroxidation results in the formation of a mixture of nonspecific stereoisomers that are highly toxic for cells [20]. More than 30 different bioactive oxidation products have been identified that accumulate in atherosclerotic lesions [21]. Degradation of lipids continues with cyclization, fragmentation and production of highly reactive terminal degradation products, such as 4-hydroxynonenal (4-HNE), malondialdehyde (MDA), acrolein, 4-hydroxy-2E,6Z-dodecadienal (4-HDDE) and others [17]. MDA gives rise to more complex lipid motifs, such as a highly reactive and immunogenic malondialdehyde-acetaldehyde (MAA). All these products can be toxic due to formation of adducts with cellular proteins by covalent reaction with amino acid residues mainly with lysine, histidine and cysteine [17].

The modification of proteins and lipids generates structural neo-epitopes that are recognized by receptors of the immune system. These neo-epitopes are also known as oxidation-specific epitopes (OSEs) [20]. OSEs can also be generated by modification of proteins with truncated phospholipids, such as oxidized phosphatidylcholine, oxidized cardiolipin (OxCL), oxidized phosphatidylserine (OxPS) and oxidized phosphatidylethanolamine (OxPE). Phosphocholine (PC)-containing oxidized phospholipid (PC-oxPL), oxidized cholesteryl ester (ox-CE), MDA and 4-HNE have all been documented in atherosclerotic lesions both in humans and in animal models [20]. The study of coronary artery sections from sudden death victims and carotid endarterectomy specimens demonstrated that OSEs were absent in normal coronary arteries and minimally present in early atherosclerotic lesions. At the same time, MDA-related epitopes and oxPLs were strongest in late lesions in macrophage-rich areas and the necrotic core, and they were specifically associated with unstable and ruptured plaques [22]. Lipid peroxidation was shown to be a significant predictor of the presence of subclinical carotid atherosclerosis in patients with type 2 diabetes mellitus [23]. 

Phospholipid oxidation products were shown to accumulate in several diseases that predispose to heart attack, including lupus and rheumatoid arthritis [21]. OxPL present on Lipoprotein(a), Lp(a), are thought to mediate, in part, the ability of Lp(a) to promote atherogenesis and aortic valve calcification [24]. They may play a causative role in the induction of thrombosis, as treatment with oxPL increased platelet activation in animal hyperlipidemic models [21]. 

Cholesteryl ester (CE) hydroperoxides were shown to be biologically active components of oxLDLs. In a model of minimally oxidized LDL (mmLDL) in which native LDL is modified by cells expressing 12/15-lipoxygenase, macrophage activation was associated with oxidized CE. OxCEs were found in the extracts of atherosclerotic lesions isolated from hyperlipidemic *apoe*^−/−^ mice [25]. The presence of oxidized cholesteryl arachidonate with bicyclic endoperoxide and hydroperoxide groups (BEP-CE) was demonstrated in human plasma and in the human plaque material [26].

One of the features of atheroinflammation is a high level of protein carbonylation. Covalent adduction of aldehydes to apolipoprotein B in LDL was shown to be strongly implicated in the mechanism of atherogenic modification of LDL [27]. A study on oxLDL lysine and histidine adductome identified Nϵ-(8-carboxyoctanyl)lysine (COL) as a major product of carbonylation in vitro. It was shown to be significantly higher in hyperlipidemic mice and atherosclerosis patients [28]. Moreover, in atherosclerosis patients, multiple MDA-Apo B adducts that resemble autoantigens recognized by antibodies were also described [29].

The subendothelial retention and oxidation of LDL also results in MDA-modifications of surrounding extracellular matrix proteins, including fibronectin, collagen type I-IV and ten ascin-C [30,31], giving rise to new potential antigens. MDA-collagen type IV-specific IgG antibodies were shown to be associated with more severe carotid disease and increased risk of myocardial infarction [32]. It was shown that LDL oxidation in the arterial wall is associated with further modification of surrounding extracellular matrix components through aldehyde formation. Among the modified proteins, fibronectin, collagen type I and III and tenascin-C were named [30]. The study reported the presence of autoantibodies to these modified proteins in human plasma, highlighting the immunogenic properties of such modifications.

## 3. Receptor-Mediated Uptake and Effects of Oxidation-Specific Epitopes

### 3.1. Interaction with CD36

Formed as a result of oxidative modification of protein and lipid moieties of LDL, OSEs can be recognized by the immune system, since they resemble the markers of oxidative stress and tissue damage. Moreover, some of the known OSEs resemble the molecular patterns of bacterial cells [20]. It was shown that OSEs were present not only on oxLDL, but also on apoptotic cells, apoptotic blebs and cellular debris. Similarly to other described damage-associated molecular patterns, OSEs are sensed by a number of pattern recognition receptors (PRRs) widely expressed on the innate immune system cells [20]. OSE sensing by PRRs mediates sterile inflammation or clearance and neutralization of OSE-exposing targets depending on the context and the receptor involved (Table 1). Macrophages are the main population of immune cells responsible for OSE sensing and clearance. 

OSE are recognized by a variety of receptors belonging to different classes: Scavenger, Toll-like receptors (TLR) and others (Table 1) [20,33]. In in vitro assays, scavenger receptors SR-A1, SR-A2 and CD36 are responsible for most of the oxLDL uptake by macrophages. Correspondingly, cells deficient for CD36, SR-A1 and SR-A2 showed a 75–90% decrease of oxLDL binding and degradation [34].

**Figure 2:**
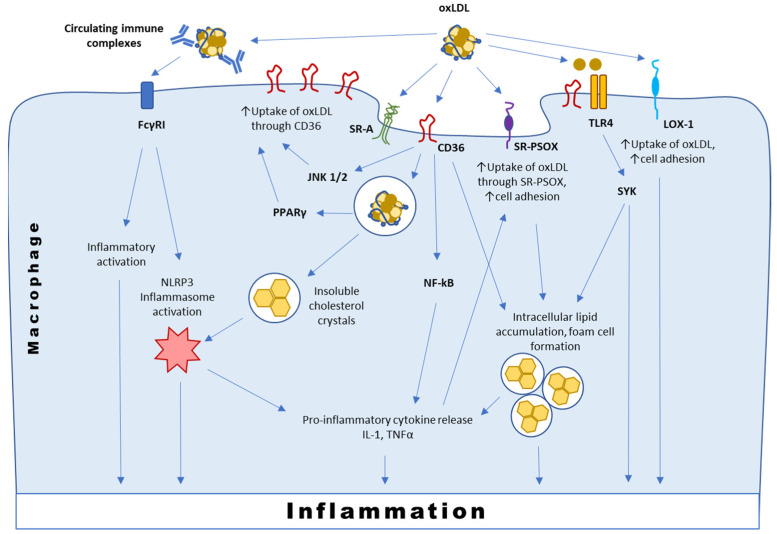
Simplified presentation of interaction of oxLDL with macrophage membrane receptors.

Among the scavenger receptors, CD36, which is the main contributor to oxLDL influx, is currently best characterized. CD36 is a pattern recognition receptor that binds polyanionic ligands that can be present both on pathogens and host cells, including thrombospondin-1, oxPLs, hexarelin, fibrillar Aβ amyloid peptides and long-chain fatty acids [35]. Genetic deletion or chemical blockade of CD36 in atherosclerosis-susceptible murine models were shown to be protective against the pathology development [36,37,38]. In addition to oxLDL recognition and transport, CD36 also has signaling functions (Figure 2). In macrophages, interaction between CD36 and oxLDL induces the phosphorylation of Lyn and the subsequent activation of the Jun kinases (JNK) 1 and 2. Signaling mediated by CD36 activates nuclear factor kappa beta (NFkB) and pro-inflammatory cytokine response that recruits the immune cells and promotes their infiltration in the arterial intima [35]. 

CD36-dependent internalization of oxLDL drives intralysosomal conversion of soluble oxLDL into insoluble cholesterol crystals that are potent activators of NLRP3-inflammasome [39]. The inflammasome is a major component of innate immunity in macrophages. Its activation is a universal response to various pathogens and mediators of sterile inflammation, which results in subsequent cleavage and activation of caspase-1 and release of IL-1β/IL-18 by monocytes/macrophages [40]. Inhibition of inflammasome activation was shown to be protective in atherosclerosis, and knocking out the inflammasome-related gene *nlrp3* in mice completely abolished the development of plaques [41]. It was shown that the expression of NLRP3 and IL-1β mRNA were significantly increased in human atherosclerotic plaques compared to unaffected arterial wall tissue, and the level of NLRP3 mRNA was higher in plaques of symptomatic patients [42]. Mechanisms of NLRP3/IL-1β regulation and involvement of the pathway in atherosclerosis pathogenesis were covered in recent reviews [43,44]. The promising concept of NLRP3/IL-1β targeting for atherosclerosis treatment was recently proven in CANTOS trial (Canakinumab Anti-Inflammatory Thrombosis Outcomes Study), which demonstrated the therapeutic potential of a monoclonal IL-1β-neutralizing antibody canakinumab in patients with prior myocardial infarction and residual inflammatory risk [45].

After internalization, oxLDL becomes a ligand for PPARγ, therefore creating a positive feedback loop, upregulating CD36 expression and facilitating further oxLDL uptake by macrophages [46,47]. Moreover, PPARγ is known to have anti-atherosclerotic functions, such as alternative macrophage polarization. However, the disruption of PPARγ negative regulation in mice leads to aggravation of atherosclerosis development [47]. This mechanism appears to be relevant for human atherosclerosis, since upregulation of PPARγ signature is a general characteristic of human atherosclerotic vessels. Analysis of laser micro-dissected macrophages from ruptured and non-ruptured carotid plaques has shown that PPARγ signaling was the most upregulated pathway in ruptured plaques with a significant increase in CD36 expression [47]. 

### 3.2. Interaction with SR-PSOX

SR-PSOX, identical to chemokine CXCL16, was also found to be involved in atherogenesis [48]. It is expressed as a transmembrane protein, but due to proteolytic cleavage, the extracellular domain is released and may circulate as a soluble chemokine important for T cell migration. SR-PSOX was shown to be a specific scavenger receptor for oxLDL, as well as adhesion molecule used by monocytes and T cells [49]. SR-PSOX-mediated uptake of oxLDL was shown to be important for foam cell formation [50]. In rabbit aorta, SR-PSOX/CXCL16 expression shifts during atherosclerosis progression from endothelial cells in early lesions and sites predisposed to plaque formation to intimal macrophages in more developed plaques. SR-PSOX is upregulated by combinations of the pro-inflammatory cytokines interferon-gamma (IFN-y) and tumor necrosis factor-alpha (TNF-α) that are major actors in atherogenesis [49]. SR-PSOX mRNA expression was shown to be prominent in human atherosclerotic lesions but undetectable in normal aortic tissue [49]. Moreover, the severity of the lesions was related to a specific polymorphism in the SR-PSOX/CXCL16 gene [51]. High levels of SR-PSOX in circulation showed a positive correlation with acute events in coronary artery disease [52].

### 3.3. Interaction with Immunoglobulins and TLRs

A large fraction of circulating modified LDL can be bound by specific antibodies, forming immune complexes. Autoantibodies can develop to various types of modified LDL, including oxLDL [53]. Presence of circulating immune complexes containing modified LDL has long been known as a risk factor of atherosclerosis progression [53,54]. A large study that included patients with type 1 diabetes revealed the strong predictive value of cholesterol and ApoB contents (used as surrogate markers of modified LDL) of circulating immune complexes for carotid intima-media thickness progression [54]. A more recent study confirmed the association of modified LDL autoantibodies with cardiovascular disease outcomes in type 1 diabetes patients and the positive effect of statin therapy on both LDL cholesterol and LDL-containing immune complexes levels, which is correlated with the cardiovascular risk reduction [55]. These observations warrant further studies of the interaction of LDL-containing immune complexes with the immune cells and the role of such interaction in atherosclerosis.

It was shown that oxLDL-containing immune complexes (oxLDL-IC) induced pro- inflammatory activation of macrophages mediated by Fc gamma receptor I (FcγRI) [15]. OxLDL-ICs may also act as a priming signal for the inflammasome (Figure 2). 

Individual oxLDL components might use TLR to activate different signaling pathways (Figure 2). TLR activation in macrophages as well as in the endothelial cells has a proatherogenic effect that was demonstrated in murine atherosclerosis models. OxPLs induces the expression of chemoattractants, such as CC-chemokine ligand 2 (CCL2), fibronectin-containing connecting segment 1, CXCL8 and P-selectin, and triggers monocyte binding to endothelial cells, which is important for monocyte infiltration into the intima. Depending on the model, these proatherogenic effects are mediated by TLR4 [20,56] or TLR2 pathways [57]. Chemokine secretion by macrophages stimulated with oxLDL requires the cooperation of CD36 with a TLR4–TLR6 heterodimer and heterotrimeric complex assembly [57]. 

Oxidized cholesteryl esters (oxCEs), such as cholesteryl (9,11)-epidioxy-15-hydroperoxy-(5Z,13E)-prostadienoate (BEP-CE), have been identified as active components of oxLDL that are formed when LDL is incubated with cells expressing 15-LO. 15-LO-modified, minimally oxidized LDL (mmLDL) does not contain advanced lipid oxidation products and does not bind to CD36. mmLDL binds to CD14, a receptor that recognizes bacterial lipopolysaccharide (LPS) and presents it to Toll-like receptor-4 (TLR4)/lymphocyte antigen 96 (MD-2) [58]. Macrophages deficient for TLR4 fail to produce inflammatory cytokines in response to mmLDL or oxCE.

Although oxLDL can bind the same TLR4/MD2 complex as LPS, the downstream signaling induced by these agents is not the same. The majority of LPS effects are mediated by the myeloid differentiation primary response 88 (MyD88) TLR4 adaptor, while the effects of mmLDL are mediated by spleen tyrosine kinase (SYK) [26,58]. SYK-dependent reactions are distinguished by smaller cytokine production, but more profound cytoskeletal responses, macropinocytosis-associated intracellular lipid accumulation and foam cell formation [58]. SYK inhibitor fostamatinib, administered preventively to *ldlr*^−/−^ or *apoe*^−/−^ mice on high-cholesterol diet was effective for reduction of monocytosis, vascular inflammation and atherosclerotic lesion size [59], but had no effect on established disease [60]. This observation may indicate that degradation of early oxidation products such as BEP-CE and reduced TLR4/SYK pathway signaling in advanced atherosclerotic plaques [58].

CEP, 2-(ω-carboxyethyl)pyrrol, represents an adduct between (E)-4-hydroxy-7-oxohept-5-enoic acid (an oxidative fragment of docosahexaenoic acid) and the amino groups of lysines or aminophospholipids [20]. Expression of TLR2 is increased in endothelial cells at sites of disturbed blood flow, and TLR2 deficiency in non-hematopoietic cells reduces atherosclerosis in *ldlr*^−/−^ mice [61]. In macrophages, CEP sensing by TLR2 results in M1-like polarization and expression of iNOS, IL-1β, TNFα and IL-12 [58]. Scavenger activity of macrophages towards oxLDL depends on their polarization, as was clearly shown for CEP: F4/80(hi) and M2-skewed macrophages were much more efficient at CEP binding and scavenging compared with F4/80(lo) and M1-skewed macrophages [62].

### 3.4. Interaction with LOX-1 and Other Scavenger Receptors

Lectin-like oxidized low-density lipoprotein receptor-1 (LOX-1) is responsible for sensing 4-HNE and MDA. It is primarily expressed by macrophages, endothelial and smooth muscle cells [63]. LOX-1 expression is very low in normal physiological state but is upregulated in vascular endothelium of human atherosclerotic plaques and ischemic tissues. Once activated, it stimulates the expression of adhesion and remodeling molecules, pro-inflammatory signaling pathways and proangiogenic proteins. Studies in Watanabe-heritable hyperlipidemic rabbits revealed that LOX-1 upregulation may in fact precede the atherosclerotic lesion formation. The role of such upregulation in early pathogenesis of atherosclerosis may be connected with endothelium activation and monocyte adhesion [64]. LOX-1 activation was described as a potential mechanism linking atherosclerosis with metabolic syndrome and cancer [63]. 

In addition to LOX-1, MDA epitopes can be recognized by the scavenger receptor SR-A1, the Fcγ receptor CD16, complement factors H and C3a, stabilin-1 and a number of natural antibodies [65]. CD16, Fc gamma receptor III normally involved in the recognition of immune complexes, also possesses scavenger receptor activity. It specifically binds MDA, inhibiting MDA recognition by other types of receptors (CD36, SR-A and LOX-1). Loss of CD16 expression can lead to reduced levels of MDA-induced proinflammatory cytokine expression [66]. Arterial lesion formation was significantly decreased in apoE-Fcγ-chain double knock-out mice [67]. The data generated in this model suggested that FcγR promoted atherosclerosis by inducing atheropathogenic Th17 responses [67]. The effect may be even more complex because a dichotomic role of FcγRIII in atherosclerosis depending on the lesion stage [68]. Differentiation of the effects mediated by direct recognition of oxLDL and interaction with oxLDL-IgG complexes can be challenging. 

The pathogenic effects of oxLDL may be enhanced due to its interaction with various proteins. OxLDL, but not native LDL, binds to β2 glycoprotein I (β2 GPI) forming oxLDL/β2GPI complexes that were described in several autoimmune diseases and are known to promote autoimmune recognition and T cell response. These complexes are also present in atherosclerotic plaques. oxLDL/β2GPI, recognized by anti β2GPI Ab, accelerates atherosclerosis development by promoting endothelial cell activation and the accumulation of lipids in macrophages and vascular smooth muscle cell [69].

### 3.5. Interaction with Soluble Factors and Chaperons

OSE can also be sensed by soluble factors: Complement factor H (CFH), C3a, C-reactive protein (CRP), Annexin A5 and others, including innate natural antibodies [16]. These interactions may lead to complement activation as well as opsonization and enhanced efferocytosis of OSE-bearing targets. CFH is the major inhibitor of the alternative pathway of complement activation that was recently shown to bind to and prevent the proinflammatory effects of MDA epitopes [70]. CRP-oxLDL interaction triggers complement activation and enhances binding of oxLDL-antibody complexes to Fcγ receptors expressed on macrophages. CRP and modified LDL are colocalized in early atherosclerotic lesions of humans with coronary artery disease [12]. Expression of CRP is a characteristic feature of M1 pro-inflammatory macrophages [71]. In atherosclerotic plaques, CRP participates in a positive feedback loop with oxLDL, whereby increased levels of oxLDL induce endothelial cells and macrophages to express CRP, which may in turn increase the expression of LOX-1 to promote the uptake of atherogenic LDL into cells [72]. Involvement of complement in the pathogenesis of atherosclerosis is being actively studied and was recently discussed elsewhere [13,14].

Interaction of oxLDL with monocytes and endothelial cells upregulates the expression of HSP60 [73,74], a molecular chaperone that is predominantly involved in mitochondrial protein folding and is increased upon mitochondrial stress. The increase of HSP60 level leads to its membrane translocation and secretion [74]. Despite being endogenous proteins, HSPs may be antigenic due to the molecular mimicry with bacterial HSPs [75]. Soluble HSP60 in the extracellular milieu triggers the inflammatory immune response in macrophages via TLR-4 activation and subsequent activation of B and T cell responses. Similarly to other HSP proteins, HSP60 is considered as potential proatherogenic protein because of several observations. First, HSP60 induces endothelial cells activation, which may be relevant for early atherosclerotic lesion formation. Atherogenic activation of endothelial cells as a result of exposure to oxLDL with upregulation of adhesion molecules, such as intercellular cell adhesion molecule-1 (ICAM-1), vascular cell adhesion molecule-1 (VCAM-1), E-selectin and chemokines (MCP-1), was clearly shown to be HSP60-dependent [74]. Second, HSP60-specific T lymphocytes are abundant in early atherosclerotic lesions in humans [76], and their presence in circulation potentiates atherosclerosis development in hyperlipidemic mouse model [75]. Furthermore, HSP60 gene polymorphisms were found to correlate with the pathological grade and incidence rate of atherosclerosis [77]. Finally, high serum levels of antibodies against HSP60 were shown to be associated with atherosclerotic diseases, such as coronary artery diseases or cerebrovascular events. The idea of tolerization with antigenic HSP60 protein or its peptides is widely studied and was covered in a recent review [78].

## 4. Anti-Inflammatory and Immunoregulatory Functions of oxLDL

The pro-inflammatory effects of oxLDL discussed above represent only one aspect of the biological effects of oxLDL. It is suggested that at physiological concentrations, oxLDL may play immunoregulatory and adaptive role, as do immunoregulatory oxPLs oxPAPC (oxidized 1-palmitoyl-2-arachidonoyl-sn-glycero-3-phosphocholine). The anti-inflammatory activities of oxPAPC as well as other oxPLs are regulated in part by the transcription factor nuclear factor (erythroid-derived 2)-like 2 (Nrf2) [79]. Components of OxPAPC induce a covalent modification of cysteine residues in Keap-1, which disrupts the Keap-1-Nrf2 interaction and leads to Nrf2 stabilization, therefore upregulating the genes of antioxidant response (HO-1, NQO1, GCL and glutathione-S-transferases) [79]. These genes are also known for their atheroprotective activity. However, the role of Nrf2 in atherosclerosis progression is not so straightforward as Nrf2 activation at later stages of the disease, and Nrf2-dependent inflammasome activation was also described [80].

Synthetic oxPL analogs, lecinoxoids, have been studied in atherosclerosis models. VB-201, a small-molecule lecinoxoid, exhibited up to 90% inhibition of monocyte chemotaxis in vitro [81]. It was found to bind directly to TLR-2 and CD14, restricting TLR2/TLR4 signaling, and has been protective in *apoe*^−/−^ mice and in a rabbit model without affecting cholesterol or triglyceride levels [58,81].

OxPE generated by 12/15-LO is a key factor in the process known as efferocytosis. Efferocytosis is clearance of apoptotic cells by professional and non-professional phagocytes, and macrophages are the main mediators of apoptotic cells clearance in atherosclerotic plaques [82]. Such clearance is important in the context of atherosclerotic plaque, since apoptotic and necrotic cell death contributes to the plaque development, forming a necrotic core in the interior of advanced plaques. In particular, the pro-apoptotic role of high concentrations of oxLDL has been described in early studies, and much effort has been invested in studying that connection [83]. Although bulk lipid accumulation promoted by high levels of oxLDL has by itself toxic effects, it also triggers signaling cascades that affect cellular metabolism and survival. For instance, recent studies have shown that in macrophages, oxLDL impacts mitochondrial function leading to cell death. Exposure to oxLDL can cause metabolic changes affecting the mitochondrial function and, ultimately, cell survival through miR-9-mediated signaling [84].

The outcomes of effective efferocytosis are prevention of secondary necrosis, termination of inflammatory responses, promotion of self-tolerance and activation of resolving pathways. When efferocytosis is impaired, these functions are compromised, leading to increased inflammation [82]. Efferocytosis is usually effective in early atherosclerotic lesions, restricting the progression of atheroinflammation, but was shown to be impaired at advanced stages, leading to the accumulation of secondarily necrotic cells in the necrotic core of atherosclerotic plaques [85]. The presence of large necrotic core is associated with vulnerable plaques that are prone to cause complications, such as myocardial infarction and stroke, and are, therefore, most dangerous [65]. OSEs may stimulate efferocytosis through interaction with MFG-E8, which binds OxPS and OxPE exposed on the surface of apoptotic cells and facilitates engulfment by phagocytes via the αvβ3 and αvβ5 integrin receptors [20]. It was shown that OxPE exposed on the surface of alternatively activated macrophages leads to sequestration of MFG-E8, favoring phagocytosis of apoptotic cells via phosphatidylserine receptors, such as T cell immunoglobulin and mucin domain 4 (TIM4), and blocking the MFG-E8-dependent uptake of cells by pro-inflammatory monocytes [20]. Thus, OSE-guided clearance of dead cells represents a physiological housekeeping function that directs the clearance to specific pathways.

## 5. OxLDL as a Macrophage Polarization Signal

In the plaque environment, macrophages are exposed to various signals and stimuli, including cytokines, modified lipids, senescent erythrocytes and hypoxia, that influence their transcriptional program and functional phenotype [11]. As a consequence, intraplaque macrophages undergo polarization to distinct subtypes playing opposite roles in atherosclerosis pathology. The early classification of macrophages to M1 (proinflammatory) and M2 (anti-inflammatory) phenotypes is currently regarded as oversimplified and outdated, but can still be useful, especially for interpreting the results of in vitro studies [86]. M1 macrophages are characterized by high production of cytokines IL-12, TNF, IL-6 and IL-1β. Proinflammatory macrophages are important for the establishment of chronic inflammatory state with impaired tissue healing [11]. They are also highly active in ROS production, which worsens intraplaque oxidative stress and tissue damage. Furthermore, M1 macrophages induce the recruitment of Th1 cells due to the expression of CXCL9, CXCL10 and CXCL5 chemokines. M1 macrophages are characterized by decreased migration activity, and their accumulation and death contribute to necrotic core formation [87]. 

Proinflammatory and tissue-damaging activities of M1 macrophages are counterbalanced by the so-called alternatively activated or M2 macrophages. They dampen the inflammatory Th1 response by producing anti-inflammatory factors IL-10, TGF-β and IL-1 receptor antagonist (IL-1Ra), which promote angiogenesis and tissue repair [11]. M2 macrophages are further subdivided into several different subclasses, but the specific roles of different M2 subpopulations in the context of atheroinflammation are not defined yet. M2 macrophages play a major role in the elimination of apoptotic cells and cell debris; they possess pro-fibrotic function and promote tissue repair. However, M2 polarization cannot be regarded as definitely atheroprotective, since it is associated with the production of pro-angiogenic factors and matrix remodeling. These processes can contribute to plaque growth and inflammation [88]. Classification of macrophages to M1/M2 subtypes does not reflect the actual diversity of macrophage phenotypes in the atherosclerotic plaque. It appears that a continuum of macrophage phenotypes exists in vivo. Moreover, macrophages retain phenotypic plasticity and may switch between different functional phenotypes according to the local cytokine milieu. Macrophage plasticity and its role in atherosclerosis are described elsewhere [87].

In atherosclerotic plaques, oxLDL is one of the key signals for macrophage polarization, which mostly promotes M1 phenotype [11]. Cholesterol crystals are responsible for the activation of NLRP3 infammasome [41], resulting in the release of IL-1 family cytokines, considered to be M1-polarizing factors. Cholesteryl esters (including 7-ketocholestery l-9 carboxynonanoate) induce M1 polarization by activating the TLR4 and nuclear factor (NF) κB signaling pathways. Intracellular accumulation of oxLDL was also shown to drive macrophage polarization towards M1 phenotype through inhibition of the transcription factor Kruppel-like factor 2 [11]. Advanced glycation end products (AGEs) were also described as M1-polarizing signals [89]. AGEs are irreversible products of the nonenzymatic glycation and oxidation of proteins, lipids and nucleic acids that activate RAGE (Receptor of AGE) signaling.

Conversely, certain lipids and their derivatives may serve as M2 macrophage polarization signals. Among such agents are 9-oxononanoyl-cholesterol, a major cholesteryl ester oxidation product [31], and resolvin D1 [11]. 

Liver X receptors (LXRs) represent an important link between cholesterol accumulation and inflammatory response in macrophages. LXRs sense cholesterol derivatives, such as oxysterols and desmosterol. LXR agonist treatment induced IL-1β expression in M1 as well as M2-polarized macrophages, and this effect was shown to be HIF-1α (Hypoxia Inducible Factor 1a)-dependent. LXR was shown both to upregulate HIF-1α expression and to stabilize HIF-1α by direct interaction with the oxygen-dependent degradation domain of HIF-1α. The exposure of macrophages to LXR agonist induced a broad transcriptional response with upregulation of several pathways connected with glycolysis and angiogenesis, known to be HIF1-regulated. Possible atheroprotective as well as atheropathogenic roles of LXR have been reviewed elsewhere [90].

Studies in mice demonstrated that in addition to M1 and M2 phenotypes, macrophages exposed to oxidized phospholipids may be switched to Mox phenotype, characterized by reduced phagocytic activity and chemotaxis. This differentiation is driven by transcription factor NFE2L2 [11]. The relevance of Mox macrophages for human pathology is unknown.

A hallmark of atherosclerotic lesions is the formation of lipid-loaden macrophages, known as foam cells. Enhanced uptake of oxLDL mediated by scavenger receptors is a prerequisite for foam cell differentiation, but conflicting evidence exists regarding the roles of individual scavenger receptors in foam cell formation [91]. Furthermore, the expression of major receptors is observed not only in macrophages, but also in aortic endothelial cells, and vascular smooth muscle cells that can also potentially transform into foam cells. Foam cell differentiation initiates various apoptotic mechanisms, including the mitochondrial pathway and proteasomal dysfunction as well as sustained cytosolic calcium accumulation. Attempts to decrease foam cell death may provide potential therapeutic strategies for slowing atherosclerosis progression at advanced stages, but they were shown to be deleterious in early lesions [91]. 

In addition to lipid uptake, foam cell formation can be influenced by levels of lipid biosynthesis and efflux with PPARs and LXRs, playing key role in these processes. LXR-α regulate transcription of ABCA1 and ABCG1, which are involved in cholesterol efflux to apoA1 and HDL, respectively. OxLDL exposure upregulates PPARγ with a decrease in the LXR-α level, resulting in enhanced accumulation of lipids. A group of CD36-activating truncated oxidized phospholipids was identified [92,93]. They included an sn-2 acyl group and incorporated a terminal γ-hydroxy(or oxo)-α,β-unsaturated carbonyl. These lipids, collectively known as oxPCCD36, were found to directly contribute to the development of foam cell formation in macrophages. Even trace amounts of oxPCCD36 were enough to induce CD36-dependent binding and LDL uptake [93]. 

Pyruvate kinase M2 (PKM2) was also found to be important for macrophage foam cell differentiation [94]. It is upregulated in oxLDL-exposed cells in a PPARγ-dependent manner and induce the Warburg effect, an increase in glycolytic flux and a decrease in oxidative phosphorylation with lactate accumulation. OxLDL also induced PKM2 interaction with HIF-1α leading to stimulation of transcription of HIF-1a-dependent genes, associated with M1 polarization. At present, PKM2 may be regarded as an attractive target for new therapeutical approaches.

## 6. Conclusions

Lipid and lipoprotein oxidation is a common pathophysiological response to oxidative stress and hyperlipidemia. Oxidized lipids and lipoproteins act as DAMPs and are sensed by a number of pattern recognition receptors. Macrophage response to oxLDL species plays an important role in atherosclerotic lesion initiation and progression. At the early stages of lesion development, it is involved in monocyte adhesion and accumulation in the vascular wall and foam cell formation. Progression of the lesion is accompanied by M1 macrophage polarization, continuous inflammatory response, involvement of the T and B cells and deficient apoptotic cells clearance, all of which can be influenced by oxLDL recognition. It is important to understand the complexity of oxLDL effects on various cell types other than macrophages, such as endothelial cells, smooth muscle and lymphocytes. Unravelling the pathways of innate and adaptive immune responses to oxLDL may highlight new possibilities for atherosclerosis prevention and treatment.

## Figures and Tables

**Table 1:** Recognition of OSEs by PRRs.

PRR	OSE	Effect	Cells
Scavenger receptors			
SR-A1,2	MDA	Uptake	Macrophages, mast, dendritic, endothelial, smooth muscle cells
SR-B1	PC-OxPL	Uptake	Monocytes/macrophages, hepatocytes, adipocytes
SRECI/II	OxLDL	Uptake	Endothelial cells, macrophages, CD8+ cells
SR-PSOX	Ox-PS	Uptake Foam cell formation	Macrophages, smooth muscle, dendritic, and endothelial cells, and B-cells and T cells
LOX-1	MDA	Monocyte adhesion Uptake Inflammation	Endothelial and smooth muscle cells, macrophages, platelets
	4-HNE		
CD36	PC-OxPL	Uptake Inflammation	Macrophages, platelets, adipocytes, epithelial and endothelial cells
	OxPS	Uptake Inflammation	
	CEP	Uptake Inflammation	
TLRs			
TLRs 4-6	PC-OxPL	Inflammation	Monocytes/macrophages, dendritic cells, mast cells, B cells
TLR4	OxCE	Inflammation Foam cell formation	Monocytes/macrophages, dendritic cells, mast cells, B cells
	OxPE	Inflammation Foam cell formation	
	4-HNE	Inflammation	
TLRs 2-6	CEP	Inflammation Thrombosis	Monocytes/macrophages, dendritic cells, mast cells, B cells, platelets
	OxPL	Angiogenesis ER stress	
TLR9	CEP	Promotion of platelet hyperreactivity and thrombosis	Platelets
Complement			
CFH	MDA	Neutralization Opsonization	
C3a	MDA	Complement activation	
CRP	PC-OxPL	Enhanced efferocytosis	
Other PRRs			
MFG-E8	OxPS	Enhanced efferocytosis	
	OxPE	Enhanced efferocytosis	
Annexin A5	OxCL	Neutralization	
CD16	MDA	Inflammation	Macrophages

Abbreviations: 4-HNE, 4-hydroxynonenal; CEP, 2-(ω-carboxyethyl) pyrrole; CFH, complement factor H; CRP, C-reactive protein; ER, endoplasmic reticulum; LOX1, lectin-like oxidized LDL receptor 1; MDA, malondialdehyde; MFG-E8, milk fat globule-epidermal growth factor 8; OSE, oxidation- specific epitope; OxCE, oxidized cholesterol esters; OxCL, oxidized cardiolipin; OxPE, oxidized phosphatidylethanolamine; OxPL, oxidized phospholipid; OxPS, oxidized phosphatidylserine; PC-OxPL, phosphocholine (PC)-containing OxPL; PRR, pattern recognition receptor; SR, scavenger receptor; TLR, Toll-like receptor.

## Data Availability

Not applicable.

## References

[1] Cinoku I.I., Mavragani C.P., Moutsopoulos H.M. (2020). Atherosclerosis: Beyond the lipid storage hypothesis. The role of autoimmunity. Eur. J. Clin. Investig..

[2] Zakiev E.R., Sukhorukov V.N., Melnichenko A.A., Sobenin I.A., Ivanova E.A., Orekhov A.N. (2016). Lipid composition of circulating multiple-modified low density lipoprotein. Lipids Health Dis..

[3] Orekhov A.N., Tertov V.V., Mukhin D.N., Mikhailenko I.A. (1989). Modification of low density lipoprotein by desialylation causes lipid accumulation in cultured cells: Discovery of desialylated lipoprotein with altered cellular metabolism in the blood of atherosclerotic patients. Biochem. Biophys. Res. Commun..

[4] Tertov V.V., Kaplun V.V., Sobenin I.A., Orekhov A.N. (1998). Low-density lipoprotein modification occurring in human plasma: Possible mechanism of in vivo lipoprotein desialylation as a primary step of atherogenic modification. Atherosclerosis.

[5] Jaakkola O., Solakivi T., Tertov V.V., Orekhov A.N., Miettinen T.A., Nikkari T. (1993). Characteristics of low-density lipoprotein subfractions from patients with coronary artery disease. Coron. Artery Dis..

[6] Tertov V.V., Kaplun V.V., Sobenin I.A., Boytsova E.Y., Bovin N.V., Orekhov A.N. (2001). Human plasma trans-sialidase causes atherogenic modification of low density lipoprotein. Atherosclerosis.

[7] Sukhorukov V.N., Karagodin V.P., Orekhov A.N. (2016). Atherogenic modification of low-density lipoproteins. Biomed. Khim..

[8] Tertov V.V., Sobenin I.A., Kaplun V.V., Orekhov A.N. (1998). Antioxidant content in low density lipoprotein and lipoprotein oxidation in vivo and in vitro. Free Radic. Res..

[9] Gianazza E., Brioschi M., Martinez Fernandez A., Casalnuovo F., Altomare A., Aldini G., Banfi C. (2021). Lipid Peroxidation in Atherosclerotic Cardiovascular Diseases. Antioxid. Redox Signal..

[10] Chinetti-Gbaguidi G., Colin S., Staels B. (2015). Macrophage subsets in atherosclerosis. Nat. Rev. Cardiol..

[11] Orekhov A.N., Nikiforov N.G., Sukhorukov V.N., Kubekina M.V., Sobenin I.A., Wu W.-K., Foxx K.K., Pintus S., Stegmaier P., Stelmashenko D. (2020). Role of Phagocytosis in the Pro-Inflammatory Response in LDL-Induced Foam Cell Formation; a Transcriptome Analysis. Int. J. Mol. Sci..

[12] Bhakdi S., Torzewski M., Klouche M., Hemmes M. (1999). Complement and atherogenesis: Binding of CRP to degraded, nonoxidized LDL enhances complement activation. Arterioscler. Thromb. Vasc. Biol..

[13] Martin-Ventura J.L., Martinez-Lopez D., Roldan-Montero R., Gomez-Guerrero C., Blanco-Colio L.M. (2019). Role of complement system in pathological remodeling of the vascular wall. Mol. Immunol..

[14] Hovland A., Jonasson L., Garred P., Yndestad A., Aukrust P., Lappegård K.T., Espevik T., Mollnes T.E. (2015). The complement system and toll-like receptors as integrated players in the pathophysiology of atherosclerosis. Atherosclerosis.

[15] Quinn M.T., Parthasarathy S., Fong L.G., Steinberg D. (1987). Oxidatively modified low density lipoproteins: A potential role in recruitment and retention of monocyte/macrophages during atherogenesis. Proc. Natl. Acad. Sci. USA.

[16] Palinski W., Hörkkö S., Miller E., Steinbrecher U.P., Powell H.C., Curtiss L.K., Witztum J.L. (1996). Cloning of monoclonal autoantibodies to epitopes of oxidized lipoproteins from apolipoprotein E-deficient mice. Demonstration of epitopes of oxidized low density lipoprotein in human plasma. J. Clin. Investig..

[17] Ito F., Sono Y., Ito T. (2019). Measurement and Clinical Significance of Lipid Peroxidation as a Biomarker of Oxidative Stress: Oxidative Stress in Diabetes, Atherosclerosis, and Chronic Inflammation. Antioxidants.

[18] Nicholls S.J., Hazen S.L. (2009). Myeloperoxidase, modified lipoproteins, and atherogenesis. J. Lipid Res..

[19] Meuwese M.C., Stroes E.S., Hazen S.L., van Miert J.N., Kuivenhoven J.A., Schaub R.G., Wareham N.J., Luben R., Kastelein J.J., Khaw K.T. (2007). Serum myeloperoxidase levels are associated with the future risk of coronary artery disease in apparently healthy individuals: The EPIC-Norfolk Prospective Population Study. J. Am. Coll. Cardiol..

[20] Binder C.J., Papac-Milicevic N., Witztum J.L. (2016). Innate sensing of oxidation-specific epitopes in health and disease. Nat. Rev. Immunol..

[21] Lee S., Birukov K.G., Romanoski C.E., Springstead J.R., Lusis A.J., Berliner J.A. (2012). Role of phospholipid oxidation products in atherosclerosis. Circ. Res..

[22] van Dijk R.A., Kolodgie F., Ravandi A., Leibundgut G., Hu P.P., Prasad A., Mahmud E., Dennis E., Curtiss L.K., Witztum J.L. (2012). Differential expression of oxidation-specific epitopes and apolipoprotein(a) in progressing and ruptured human coronary and carotid atherosclerotic lesions. J. Lipid Res..

[23] Alharby H., Abdelati T., Rizk M., Youssef E., Moghazy K., Gaber N., Yafei S. (2019). Association of lipid peroxidation and interleukin-6 with carotid atherosclerosis in type 2 diabetes. Cardiovasc. Endocrinol. Metab..

[24] Que X., Hung M.Y., Yeang C., Gonen A., Prohaska T.A., Sun X., Diehl C., Määttä A., Gaddis D.E., Bowden K. (2018). Oxidized phospholipids are proinflammatory and proatherogenic in hypercholesterolaemic mice. Nature.

[25] Harkewicz R., Hartvigsen K., Almazan F., Dennis E.A., Witztum J.L., Miller Y.I. (2008). Cholesteryl ester hydroperoxides are biologically active components of minimally oxidized low density lipoprotein. J. Biol. Chem..

[26] Choi S.H., Yin H., Ravandi A., Armando A., Dumlao D., Kim J., Almazan F., Taylor A.M., McNamara C.A., Tsimikas S. (2013). Polyoxygenated cholesterol ester hydroperoxide activates TLR4 and SYK dependent signaling in macrophages. PLoS ONE.

[27] Shibata T., Shimizu K., Hirano K., Nakashima F., Kikuchi R., Matsushita T., Uchida K. (2017). Adductome-based identification of biomarkers for lipid peroxidation. J. Biol. Chem..

[28] Uchida K., Shibata T., Toyokuni S., Daniel B., Zarkovic K., Zarkovic N., Sasson S. (2018). Development of a novel monoclonal antibody against 4-hydroxy-2E,6Z-dodecadienal (4-HDDE)-protein adducts: Immunochemical application in quantitative and qualitative analyses of lipid peroxidation in vitro and ex vivo. Free Radic. Biol. Med..

[29] Fredrikson G.N., Hedblad B., Berglund G., Alm R., Ares M., Cercek B., Chyu K.Y., Shah P.K., Nilsson J. (2003). Identification of immune responses against aldehyde-modified peptide sequences in apoB associated with cardiovascular disease. Arterioscler. Thromb. Vasc. Biol..

[30] Duner P., To F., Alm R., Gonçalves I., Fredrikson G.N., Hedblad B., Berglund G., Nilsson J., Bengtsson E. (2009). Immune responses against fibronectin modified by lipoprotein oxidation and their association with cardiovascular disease. J. Intern. Med..

[31] Vallejo J., Dunér P., To F., Engelbertsen D., Gonçalves I., Nilsson J., Bengtsson E. (2019). Activation of immune responses against the basement membrane component collagen type IV does not affect the development of atherosclerosis in ApoE-deficient mice. Sci. Rep..

[32] Vallejo J., Dunér P., Fredrikson G.N., Nilsson J., Bengtsson E.J. (2017). Autoantibodies against aldehyde-modified collagen type IV are associated with risk of development of myocardial infarction. Intern. Med..

[33] Leibundgut G., Witztum J.L., Tsimikas S. (2013). Oxidation-specific epitopes and immunological responses: Translational biotheranostic implications for atherosclerosis. Curr. Opin. Pharmacol..

[34] Kunjathoor V.V., Febbraio M., Podrez E.A., Moore K.J., Andersson L., Koehn S., Rhee J.S., Silverstein R., Hoff H.F., Freeman M.W. (2002). Scavenger receptors class A-I/II and CD36 are the principal receptors responsible for the uptake of modified low density lipoprotein leading to lipid loading in macrophages. J. Biol. Chem..

[35] Park Y.M. (2014). CD36, a scavenger receptor implicated in atherosclerosis. Exp. Mol. Med..

[36] Kuchibhotla S., Vanegas D., Kennedy D.J., Guy E., Nimako G., Morton R.E., Febbraio M. (2008). Absence of CD36 protects against atherosclerosis in ApoE knock-out mice with no additional protection provided by absence of scavenger receptor A I/II. Cardiovasc. Res..

[37] Marleau S., Harb D., Bujold K., Avallone R., Iken K., Wang Y., Demers A., Sirois M.G., Febbraio M., Silverstein R.L. (2005). EP 80317, a ligand of the CD36 scavenger receptor, protects apolipoprotein E-deficient mice from developing atherosclerotic lesions. FASEB J..

[38] Bujold K., Mellal K., Zoccal K.F., Rhainds D., Brissette L., Febbraio M., Marleau S., Ong H. (2013). EP 80317, a CD36 selective ligand, promotes reverse cholesterol transport in apolipoprotein E-deficient mice. Atherosclerosis.

[39] Sheedy F.J., Grebe A., Rayner K.J., Kalantari P., Ramkhelawon B., Carpenter S.B., Becker C.E., Ediriweera H.N., Mullick A.E., Golenbock D.T. (2013). CD36 coordinates NLRP3 inflammasome activation by facilitating intracellular nucleation of soluble ligands into particulate ligands in sterile inflammation. Nature Immunol..

[40] Paramel Varghese G., Folkersen L., Strawbridge R.J., Halvorsen B., Yndestad A., Ranheim T., Krohg-Sørensen K., Skjelland M., Espevik T., Aukrust P. (2016). NLRP3 Inflammasome Expression and Activation in Human Atherosclerosis. J. Am. Heart Assoc..

[41] Duewell P., Kono H., Rayner K.J., Sirois C.M., Vladimer G., Bauernfeind F.G., Abela G.S., Franchi L., Nuñez G., Schnurr M. (2010). NLRP3 inflammasomes are required for atherogenesis and activated by cholesterol crystals. Nature.

[42] Grebe A., Hoss F., Latz E. (2018). NLRP3 Inflammasome and the IL-1 Pathway in Atherosclerosis. Circ. Res..

[43] Hoseini Z., Sepahvand F., Rashidi B., Sahebkar A., Masoudifar A., Mirzaei H. (2018). NLRP3 inflammasome: Its regulation and involvement in atherosclerosis. J. Cell. Physiol..

[44] Liaqat A., Asad M., Shoukat F., Khan A.U. (2020). A Spotlight on the Underlying Activation Mechanisms of the NLRP3 Inflammasome and its Role in Atherosclerosis: A Review. Inflammation.

[45] Ridker P.M., Everett B.M., Thuren T., MacFadyen J.G., Chang W.H., Ballantyne C., Fonseca F., Nicolau J., Koenig W., Anker S.D. (2017). Antiinflammatory Therapy with Canakinumab for Atherosclerotic Disease., CANTOS Trial Group. N. Engl. J. Med..

[46] Tontonoz P., Nagy L., Alvarez J.G., Thomazy V.A., Evans R.M. (1998). PPARgamma promotes monocyte/macrophage differentiation and uptake of oxidized LDL. Cell.

[47] Oppi S., Nusser-Stein S., Blyszczuk P., Wang X., Jomard A., Marzolla V., Yang K., Velagapudi S., Ward L.J., Yuan X.M. (2020). Macrophage NCOR1 protects from atherosclerosis by repressing a proatherogenic PPARgamma signature. Eur. Heart J..

[48] Hofnagel O., Engel T., Severs N.J., Robenek H., Buers I. (2011). SR-PSOX at sites predisposed to atherosclerotic lesion formation mediates monocyte-endothelial cell adhesion. Atherosclerosis.

[49] Minami M., Kume N., Shimaoka T., Kataoka H., Hayashida K., Akiyama Y., Nagata I., Ando K., Nobuyoshi M., Hanyuu M. (2001). Expression of SR-PSOX, a novel cell-surface scavenger receptor for phosphatidylserine and oxidized LDL in human atherosclerotic lesions. Arterioscler. Thromb. Vasc. Biol..

[50] Zhang L., Liu H.J., Li T.J., Yang Y., Guo X.L., Wu M.C., Rui Y.C., Wei L.X. (2008). Lentiviral vector-mediated siRNA knockdown of SR-PSOX inhibits foam cell formation in vitro. Acta Pharmacol. Sin..

[51] Lundberg G.A., Kellin A., Samnegård A., Lundman P., Tornvall P., Dimmeler S., Zeihze A.M., Hamsten A., Hansson G.K., Eriksson P. (2005). Severity of coronary artery stenosis is associated with a polymorphism in the CXCL16/SR-PSOX gene. J. Intern. Med..

[52] Sun Y., Chang Z., Zhang S. (2008). Increased serum CXCL16 level is a marker for acute coronary syndromes. Arch. Med. Res..

[53] Tertov V.V., Sobenin I.A., Orekhov A.N., Jaakkola O., Solakivi T., Nikkari T. (1996). Characteristics of low density lipoprotein isolated from circulating immune complexes. Atherosclerosis.

[54] Lopes-Virella M.F., McHenry M.B., Lipsitz S., Yim E., Wilson P.F., Lackland D.T., Lyons T., Jenkins A.J., Virella G., DCCT/EDIC Research Group (2007). Immune complexes containing modified lipoproteins are related to the progression of internal carotid intima-media thickness in patients with type 1 diabetes. Atherosclerosis.

[55] Lopes-Virella M.F., Bebu I., Hunt K.J., Virella G., Baker N.L., Braffett B., Gao X., Lachin J.M., DCCT/EDIC Research Group (2019). Immune Complexes and the Risk of CVD in Type 1 Diabetes. Diabetes.

[56] Imai Y., Kuba K., Neely G., Yaghubian-Malhami R., Perkmann T., van Loo G., Ermolaeva M., Veldhuizen R., Leung Y.H.C., Wang H. (2008). Identification of oxidative stress and Toll-like receptor 4 signaling as a key pathway of acute lung injury. Cell.

[57] Kadl A., Sharma P.R., Chen W., Agrawal R., Meher A.K., Rudraiah S., Grubbs N., Sharma R., Leitinger N. (2011). Oxidized phospholipid-induced inflammation is mediated by Toll-like receptor 2. Free Radic. Biol. Med..

[58] Mendel I., Feige E., Yacov N., Salem Y., Levi I., Propheta-Meiran O., Shoham A., Ishai E., George J., Harats D. (2014). VB-201, an oxidized phospholipid small molecule, inhibits CD14- and Toll-like receptor-2-dependent innate cell activation and constrains atherosclerosis. Clin. Exp. Immunol..

[59] Hilgendorf I., Eisele S., Remer I., Schmitz J., Zeschky K., Colberg C., Stachon P., Wolf D., Willecke F., Buchner M. (2011). The oral spleen tyrosine kinase inhibitor fostamatinib attenuates inflammation and atherogenesis in low-density lipoprotein receptor-deficient mice. Arterioscler. Thromb. Vasc. Biol..

[60] Lindau A., Härdtner C., Hergeth S.P., Blanz K.D., Dufner B., Hoppe N., Anto-Michel N., Kornemann J., Zou J., Gerhardt L.M. (2016). Atheroprotection through SYK inhibition fails in established disease when local macrophage proliferation dominates lesion progression. Basic Res. Cardiol..

[61] Mullick A.E., Soldau K., Kiosses W.B., Bell T.A., Tobias P.S., Curtiss L.K. (2008). Increased endothelial expression of Toll-like receptor 2 at sites of disturbed blood flow exacerbates early atherogenic events. J. Exp. Med..

[62] Kim Y.W., Yakubenko V.P., West X.Z., Gugiu G.B., Renganathan K., Biswas S., Gao D., Crabb J.W., Salomon R.G., Podrez E.A. (2015). Receptor-Mediated Mechanism Controlling Tissue Levels of Bioactive Lipid Oxidation Products. Circ. Res..

[63] Balzan S., Lubrano V. (2018). LOX-1 receptor: A potential link in atherosclerosis and cancer. Life Sci..

[64] Chen H., Li D., Sawamura T., Inoue K., Mehta J.L. (2000). Upregulation of LOX-1 expression in aorta of hypercholesterolemicrabbits: Modulation by losartan. Biochem. Biophys. Res. Commun..

[65] Hansson G.K., Libby P., Tabas I. (2015). Inflammation and plaque vulnerability. J. Intern. Med..

[66] Zhu X., Ng H.P., Lai Y., Craigo J.K., Nagilla P.S., Raghani P., Nagarajan S. (2014). Scavenger receptor function of mouse Fcgamma receptor III contributes to progression of atherosclerosis in apolipoprotein E hyperlipidemic mice. J. Immunol..

[67] Ng H.P., Burris R.L., Nagarajan S. (2011). Attenuated atherosclerotic lesions in apoE-Fcγ-chain-deficient hyperlipidemic mouse model is associated with inhibition of Th17 cells and promotion of regulatory T cells. J. Immunol..

[68] Asare Y., Koehncke J., Selle J., Simsekyilmaz S., Jankowski J., Shagdarsuren G., Gessner J.E., Bernhagen J., Shagdarsuren E. (2020). Differential Role for Activating FcγRIII in Neointima Formation After Arterial Injury and Diet-Induced Chronic Atherosclerosis in Apolipoprotein E-Deficient Mice. Front. Physiol..

[69] Zhang G., Cai Q., Zhou H., He C., Chen Y., Zhang P., Wang T., Xu L. (2021). OxLDL/beta2GPI/anti-beta2GPI Ab complex induces inflammatory activation via the TLR4/NF-kappaB pathway in HUVECs. J. Mol. Med. Rep..

[70] Weismann D., Hartvigsen K., Lauer N., Bennett K.L., Scholl H.P., Charbel Issa P., Cano M., Brandstätter H., Tsimikas S., Skerka C. (2011). Complement factor H binds malondialdehyde epitopes and protects from oxidative stress. Nature.

[71] Kaplan M., Shur A., Tendler Y. (2018). M1 Macrophages but Not M2 Macrophages Are Characterized by Upregulation of CRP Expression via Activation of NFkappaB: A Possible Role for Ox-LDL in Macrophage Polarization. Inflammation.

[72] Stancel N., Chen C.C., Ke L.Y., Chu C.S., Lu J., Sawamura T., Chen C.H. (2016). Interplay between CRP, Atherogenic LDL, and LOX-1 and Its Potential Role in the Pathogenesis of Atherosclerosis. Clin. Chem..

[73] Frostegard J., Kjellman B., Gidlund M., Andersson B., Jindal S., Kiessling R. (1996). Induction of heat shock protein in monocytic cells by oxidized low density lipoprotein. Atherosclerosis.

[74] Shirsath K., Joshi A., Vohra A., Devkar R. (2021). HSP60 knockdown exerts differential response in endothelial cells and monocyte derived macrophages during atherogenic transformation. Sci. Rep..

[75] Ayada K., Yokota K., Kobayashi K., Shoenfeld Y., Matsuura E., Oguma K. (2009). Chronic infections and atherosclerosis. Clin. Rev. Allergy Immunol..

[76] Almanzar G., Ollinger R., Leuenberger J., Onestingel E., Rather B., Zehm S., Cardini B., van der Zee R., Grundtman C., Wick G. (2012). Autoreactive HSP60 epitope-specific T-cells in early human atherosclerotic lesions. J. Autoimmunol..

[77] Liao B.H., Xu Z.L., Gao F., Zhang S.H., Liang R.J., Dong S.H. (2020). The relationship between HSP60 gene polymorphisms and susceptibility to atherosclerosis. Eur. Rev. Med. Pharmacol. Sci..

[78] Wick C. (2016). Tolerization against atherosclerosis using heat shock protein 60. Cell Stress Chaperones.

[79] Mimura J., Itoh K. (2015). Role of Nrf2 in the pathogenesis of atherosclerosis. Free Radic. Biol. Med..

[80] Kadl A., Meher A.K., Sharma P.R., Lee M.Y., Doran A.C., Johnstone S.R., Elliott M.R., Gruber F., Han J., Chen W. (2010). Identification of a novel macrophage phenotype that develops in response to atherogenic phospholipids via Nrf2. Circ. Res..

[81] Feige E., Yacov N., Salem Y., Levi I., Mendel I., Propheta-Meiran O., Shoham A., Hait-Darshan R., Polonsky O., George J. (2013). Inhibition of monocyte chemotaxis by VB-201, a small molecule lecinoxoid, hinders atherosclerosis development in ApoE^−/−^ mice. Atherosclerosis.

[82] Doran A.C., Yurdagul A., Tabas I. (2020). Efferocytosis in health and disease. Nat. Rev. Immunol..

[83] Martinet W., Kockx M.M. (2001). Apoptosis in atherosclerosis: Focus on oxidized lipids and inflammation. Curr. Opin. Lipidol..

[84] Xin T., Lu C., Zhang J., Wen J., Yan S., Li C., Zhang F., Zhang J. (2020). Oxidized LDL Disrupts Metabolism and Inhibits Macrophage Survival by Activating a miR-9/Drp1/Mitochondrial Fission Signaling Pathway. Oxid. Med. Cell. Longev..

[85] Schrijvers D.M., De Meyer G.R., Kockx M.M., Herman A.G., Martinet W. (2005). Phagocytosis of apoptotic cells by macrophages is impaired in atherosclerosis. Arterioscler. Thromb. Vasc. Biol..

[86] Gordon S., Taylor P.R. (2005). Monocyte and macrophage heterogeneity. Nat. Rev. Immunol..

[87] Jinnouchi H., Guo L., Sakamoto A., Torii S., Sato Y., Cornelissen A., Kuntz S., Paek K.H., Fernandez R., Fuller D. (2020). Diversity of macrophage phenotypes and responses in atherosclerosis. Cell. Mol. Life Sci..

[88] Bi Y., Chen J., Hu F., Liu J., Li M., Zhao L. (2019). M2 Macrophages as a Potential Target for Antiatherosclerosis Treatment. Neural Plast..

[89] Han X., Ma W., Zhu Y., Sun X., Liu N. (2020). Advanced glycation end products enhance macrophage polarization to the M1 phenotype via the HIF-1alpha/PDK4 pathway. Mol. Cell. Endocrinol..

[90] Ménégaut L., Thomas C., Jalil A., Julla J.B., Magnani C., Ceroi A., Basmaciyan L., Dumont A., Le Goff W., Mathew M.J. (2020). Interplay between Liver X Receptor and Hypoxia Inducible Factor 1alpha Potentiates Interleukin-1beta Production in Human Macrophages. Cell Rep..

[91] Maguire E.M., Pearce S.W.A., Xiao Q. (2019). Foam cell formation: A new target for fighting atherosclerosis and cardiovascular disease. Vasc. Pharmacol..

[92] Podrez E.A., Poliakov E., Shen Z., Zhang R., Deng Y., Sun M., Finton P.J., Shan L., Gugiu B., Fox P.L. (2002). Identification of a novel family of oxidized phospholipids that serve as ligands for the macrophage scavenger receptor CD36. J. Biol. Chem..

[93] Serbulea V., DeWeese D., Leitinger N. (2017). The effect of oxidized phospholipids on phenotypic polarization and function of macrophages. Free Radic. Biol. Med..

[94] Kumar A., Gupta P., Rana M., Chandra T., Dikshit M., Barthwal M.K. (2020). Role of pyruvate kinase M2 in oxidized LDL-induced macrophage foam cell formation and inflammation. J. Lipid Res..

